# Bridging the Gap of the Afri–Eurocentric Worldview Divide in a Postcolonial South Africa

**DOI:** 10.3390/ijerph19031165

**Published:** 2022-01-21

**Authors:** Sharon Johnson, Izanette Van Schalkwyk

**Affiliations:** 1 Faculty of Humanities, Psychology Department, Cornerstone Institute, Cape Town 7441, South Africa; 2 Africa Unit for Transdisciplinary Health Research, Faculty of Health Sciences, North-West University, Potchefstroom 2526, South Africa; 20977026@nwu.ac.za

**Keywords:** Africentric, Eurocentric, worldview, well-being, social injustices

## Abstract

Background: This paper is an attempt to bridge the gap between Africentric and Eurocentric worldviews through the lens of positive psychology’s second wave of attaining pathways to well-being. Methods: The overcoming of existential suffering with indigenous understandings has been addressed through photo-elicitation in retrospective timelines with students Lihile+, Tanaka+, and Diana+, +Pseudonyms to protect identity Thematic analysis with semi-structured virtual interviews has also been utilized to gain insights into Africentric and Eurocentric worldviews. All students come from different contexts of cultural complexity. Lihile was raised by her maternal Xhosa family, with a traditional Sotho father. Tanaka is Shona, born and schooled in Zimbabwe, studying in South Africa. Diana was born in England and is now living in rural KwaZulu-Natal. Findings: Students’ worldviews were shaped by their primary caregivers’ multicultural influences, as well as their exposure to educational and religious contact zones. Despite having to survive the traumatic legacy of social injustices, the students managed to pursue positive goals and transcend challenges and achieve well-being. Conclusions: This study attempted to transcend the divide of Afri–Eurocentric worldviews towards a shared responsibility to develop an improved social science in Africa. Positive psychology offered a space to accommodate well-being as a healing process, not only for the oppressed but also the oppressors of past social injustices.

## 1. Introduction

This study aimed to gain insights into bridging the gap between Africentric and Eurocentric worldviews through positive psychology’s second wave of well-being, arising from the meanings of existential suffering and indigenous understandings [1]. An African way of being is central to an Africentric theory of human development in Africa, combining the interdependent, interpenetrating, and complementary planes of the material and the spiritual [2]. However, much research shows the colonial origins of African scholarship and its primarily western Eurocentric epistemology [3]. The hegemony of a Eurocentric psychology in Africa has been challenged [4]. Indeed, the present junction between Africentric and Eurocentric worldviews needs critical exploration and expression [2]. Increasingly, African scholars raise the question: “How can we put the ‘African’ back into African studies?” [5]: This point of departure entails more than the respectful cultural adapting of research conducted in, for example, South Africa (SA) and the African context [4].

As a philosophy of life and conception of the world, the African worldview has been distinguished from others “in so far as it is grounded in and grows out of African history and culture” [6]. It is holistic and humanistic, and it focuses on the universe and all the people within it as an interconnected whole, seeking harmony and rhythm [7].

The diversities inherent in African philosophy have recently been explored from both Africentric and Eurocentric worldviews [2,3,4,5]. Resorting to an ethno-philosophical approach to African epistemology and research is most preferred [3]. For example, Ebersöhn et al. [8] recommend that psychology should accommodate local (non-Western) contexts by emphasizing “the cultural context in which psychological phenomena occurs” (p. 4) to promote people’s well-being. Other scholars, such as, Nyoni [9] advocate the “setting free of those trapped within the African caged colonial mentality” (p. 4); and “decolonizing the African colonial caged mind” (p. 5). This means that postcolonial states have specific challenges regarding pedagogy as well as regarding those engaging in the social sciences [9].

The African view is based on a holistic and anthropocentric ontology, referring to a oneness with the cosmos, including God, nature, and human beings in a person-centered society [2]. The Zulu philosophy of *ubuntu* or the Sotho *batho* implies that a person is only a person because of other people.

Understandably, the complexities of current postcolonial and neocolonial challenges should not be denied, with efforts needed to re-conscientize and re-Africanize a de-Africanized continent, prioritizing people [2]. Added to the impact of collective trauma [10] through Eurocentric practices such as slavery and colonization, SA also has a history of apartheid and is a deeply divided, violent, and racist modern society [3,4]. As one of the most unequal countries economically in the world [11], added challenges such as the COVID-19 pandemic push these inequalities to new levels.

It is of paramount importance to consider contextual challenges and existential dilemmas to protect and promote well-being; and particularly to optimize positive mental health in meeting deepest human needs. This stance is integral to the second wave of positive psychology (PP 2.0).

### 1.1. Positive Psychology

Positive Psychology (PP) research and interventions were initially directed to optimize well-being (healthy functioning) and happiness (subjective well-being) that occur in ordinary living and supportive territories [1,12]. Since 2010, the second wave of PP 2.0 emerged to accommodate the existential universal, on the one hand and indigenous cultural expression, on the other. These two pillars of PP 2.0 existential positive psychology [1] and indigenous psychology [12] are complementary to each other, resulting in a greater depth of the existential dimension [13] and greater breadth of the indigenous view of happiness [14].

So, although suffering is universal, human existence also entails flourishing and high levels of well-being, with positive emotions offering valuable wellsprings [15]. Both flourishing and suffering are integral to the meaning in life, which is linked to humans’ capacity towards connectivity, referring to relational connectedness, religious practices, beliefs, and the spiritual capacity to transcend the self [12] and reach one’s full potential. Focusing on the African context, Abdulla [16] refers to the relevance of the interconnection between culture, religion and freedom of religious belief to the history of development.

Religion and spirituality have been indirectly correlated with economic development—the richer the country, the more godless it tends to be [17]. SA has a high (80) percentage of the population following the Christian faith (17). In considering the relationship between status and well-being, Berkessel [17] found that religion tends to lessen the effects of poverty on mental health. This could be attributed to the church’s sceptical attitude towards wealth as a pathway to spirituality or the social-support network that it offers worshippers [17].

### 1.2. Afrocentric Versus Africentric

The more commonly used term ‘Afrocentricity’ originated in United States of America (USA), attempting to bind together the various elements of both African and African-American studies into a unified discipline [18]. It was not new, having been associated with anti-oppression movements in the 1960s and 1970s. Africentric was first used by Nwoye in 2015—a Nigerian researcher now in SA—to emphasize “… the distinctive contributions of African culture and tradition in the making of human personhood” (p. 43) [2].

In alignment with Nwoye’s point of departure, the term Africentric is used in this paper. Since this is strongly associated with a specific context, namely Africa, we chose to use the bioecological theory of Bronfenbrenner [19,20] for human development. This theory also considers the proximal processes (third phase) involving not only relationships among people but also relations between people and the objects and symbols with which they come into contact, as well as the role of time (chronosystem).

### 1.3. A Global Generation

While the Global South research context has been interpreted as critical, postcolonial, and indeed anti-imperialist, it is acknowledged that this does not just signify a specific geographical space, but rather a unique knowledge system [21]. This could be defined by “its negative and repairing relationship with colonialism and transnational capitalism, associated with the Global North” (p. 4) [21]. Philips [22] argues that it will be more correct to use the term ‘a global generation’ for studies about contemporary youth in a postcolonial world. This is important, since most youths live in the Global South [22].

Obviously, we need to pay attention to “the enormous diversity of what youth and generations may mean today across the globe, and be reflexive of how knowledge is produced about them” (p. 1) [22]. Sibani [23] acknowledges that all cultures change through time—none are static—and the western culture has tremendously impacted on African traditional society in positive and negative dimensions. Issues include language, marriage, mode of dress, arts and crafts, food, religion, education, and social life.

In summary, the research objective was to find ways to bridge the gap between Africentric and Eurocentric worldviews, with indigenous understandings of the well-being processes needed to transcend suffering. A creative art-based method fitted well into this paradigm.

## 2. Research Design and Methods

This qualitative research used a social constructivist lens, since interactions are outcomes between individuals and their environments [23]. This provided a framework to delve into the intercultural experiences typical in postapartheid SA, where students are influenced by highly diverse and hybrid cultural backgrounds of various contact zones (CZs). To improve the accessibility of scholarship and enhance understanding of the complexities of attaining well-being, a primarily visual arts method of photo-elicitation was used, drawing on alternative and representational forms of enquiry [24,25].

Photographs allowed participants to communicate non-verbally, accessing different memories and emotions, providing insights from older evolutionary parts of the brain than verbal expression [26] Timeline research offered the scope to link experiences to the wider social, environmental, and political context [27].

Once the timeline exercise had been completed, students submitted slides virtually and the primary researcher conducted semi-structured interviews around each time phase. A secondary mixed qualitative methodology of thematic analysis was also used to deepen insights around their life experiences.

### 2.1. Participants

Three students at a higher education non-profit institute in the Western Cape took part in this small-scale study. Through selective sampling, two young black African women represented the Africentric worldview. They both attended lectures in Theories of Personality in the primary author’s third-year psychology class, expressing interest in African psychology. Participant 1, Lihile, was a 23-year-old female Bachelor of Arts (psychology) undergraduate student. She was single with no children. She was born in De Aar, a rural farming community in the Norther Cape, and speaks Xhosa, English, and Afrikaans. After attended local primary and secondary schools, she moved to Cape Town and registered to study for her psychology degree. She described herself as Christian, belonging to the Apostolic Faith Ministries. She planned to continue her studies to postgraduate level and to work in the mental health field.

Participant 2, Tanaka, was a 24-year-old psychology student in the third year of her Bachelor of Arts degree. She was single with no children. She was Shona, born in Murehwa, Zimbabwe, outside the capital Harare. She spoke Shona, Xhosa, and English. She attended five primary schools and two high schools in Zimbabwe and one in SA, before studying at university. She identified with being a Christian, belonging to the Apostolic Faith and Mission Church, but was also influenced by Jehovah Witness, Methodist, and Roman Catholic churches. She wanted to continue to Honors with her psychology studies and hoped to teach English abroad.

Participant 3, Diana, was a mature 62-year-old English woman brought up in SA, representing the Eurocentric worldview. She attended the first author’s Theories of Personality postgraduate Honors class. She grappled with her multicultural background, speaking English, Afrikaans, and isiZulu. She was married with three daughters. She attended junior schools in South Shields, England, and in East Rand, Gauteng, and two SA high schools. She studied at several tertiary institutions, including Trinity College, London, graduating in English and dramatic arts, and nursing at Grey’s Hospital in SA. She was majoring in counseling communication and hoped, through the medium of dramatic arts, to facilitate group therapy, possibly in the field of trauma counseling.

### 2.2. Data Collection and Analysis

Data were gathered using a demographic questionnaire (Table 1), photo-elicitation with visual and and/or written timeline exercise (Figure 1, Figure 2, Figure 3, Figure 4, Figure 5, Figure 6, Figure 7, Figure 8, Figure 9, Figure 10 and Figure 11) and semi-structured, individual interviews (via virtual meetings). Participant-driven photo-elicitation allowed the participants to both chose the photographs and create the dialogue of the image [28].

Retrospective timeline exercises [27,29,30] included participants’ cultural background and influences during developmental life phases and their experience in various CZs [31]. Semi-structured interviews aided in creating consistency across all the participants. The open-ended and additional probing questions enabled the collection of rich data that included thorough descriptions of students’ intra- and interpersonal intercultural experiences. They were invited to respond using their own words and to tell their personal stories within the African context to gain understandings of each worldview. Questions included: What are your primary cultural influences? Explain your value and beliefs.

The first author used NVivo 12 (Mac) software (QSR International, Melbourne, Australia) for qualitative analysis. Data analysis began with a word count. Thematic analysis was used for timeline semi-structured interview transcripts. Braun and Clarke’s five-step model [32] of analyzing data was followed. The co-author discussed the integrity and accuracy of the codes and themes. Each researcher examined the transcripts and generated the codes separately and various discussions took place toward consensus to reconcile the themes. This process of intersubjective validation was followed to yield rich, complex, and detailed descriptions and interpretations [32].

### 2.3. Ethical Considerations

Ethical approval was provided by the ethics committee of the Research Committee of the Cornerstone Institute in Cape Town, (ethics number: CNR0604212). Participants were contacted via email by the first author, who explained the purpose of the research. Appropriate ethical principles were applied to ensure protection. Students gave consent voluntarily and agreed to have their narrations published in a journal. All participants understood what was being asked of them [33], and all parties involved were competent to consent to the use of their transcripts, which were read and checked by each student. Students gave permission for the reproduction of their submitted photographic and written slide material.

Anonymity and confidentiality were assured by the omission of participant names, using pseudonyms. Recordings, transcripts, and any other data were stored on password-protected devices. For cross-checking purposes and intersubjective validation [33], transcripts were shared amongst the authors, discussed, analyzed, and interpreted. Data will be destroyed in accordance with the University’s research ethics procedure.

Qualitative research criteria, such as credibility, transferability, and dependability [34] were applied. The authors adhered to qualitative criteria to allow the replicability of procedures and data generated under different circumstances [32]. Reflexivity offered trustworthiness [34], with different researcher positions considered [35].

### 2.4. Reflexivity

As primary author, my ancestors came to Africa from England with the 1820 Settlers. I was born in rural Zimbabwe in the 1950s, experiencing the country moving from being a colony of England to declaring independence (1965) and subsequently handing governance to a Shona leader, Robert Mugabe in 1980, after a protracted civil war. I studied at Durban University in KwaZulu Natal (KZN) in SA in the 1970s, learning about apartheid injustices in political science from Professor Rick Turner, who was subsequently banned from teaching and shot by the SA Nationalist Government. As counselor and researcher on wellness in contexts of SA high-risk schools, I am mindful of the significance of my cultural positioning in these multicultural contexts.

As secondary author, I am acutely aware of different types of researcher positions [35], and I consider my role as somewhere on the continuum between an ‘insider’ and ‘outsider’ with a good understanding of the SA context. Many changes since 1976 (when my first child was born), involved reflection, especially on my past and where I was situated within the notions of social justice and apartheid. I experienced my personal years of struggle and after 1994 it became easier to ‘walk the talk’ and interact with others in a variety of CZs and multiracial activities.

## 3. Findings

**Figure 1:**
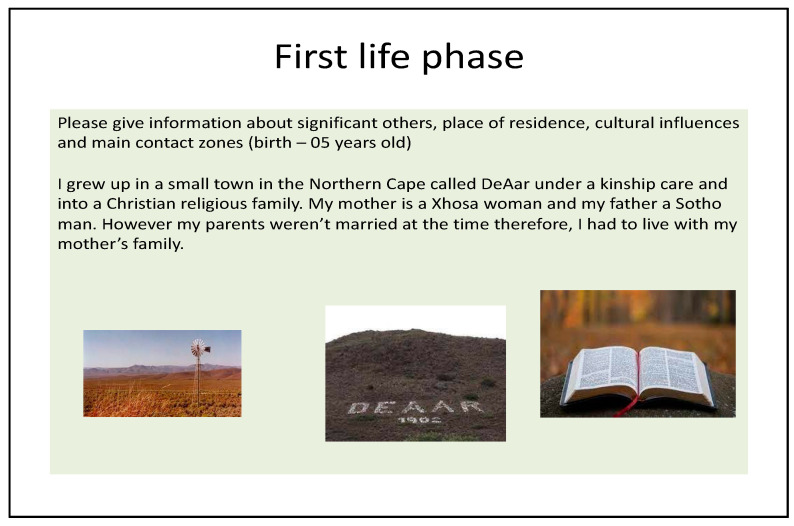
Lihile’s first life-phase timeline.

**Figure 2:**
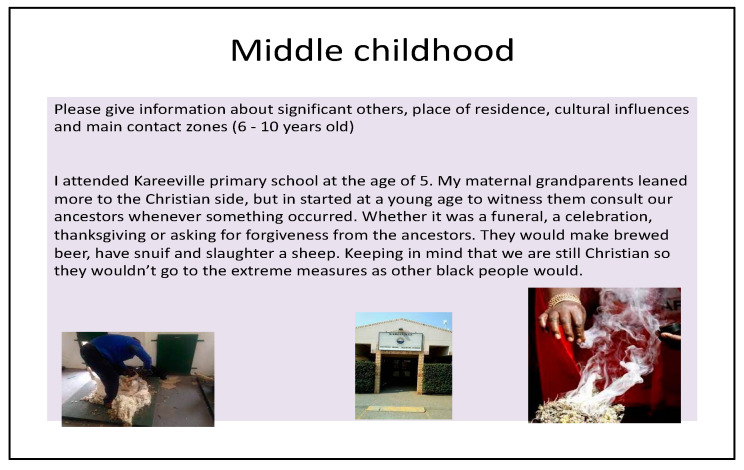
Lihile’s early childhood phase.

**Figure 3:**
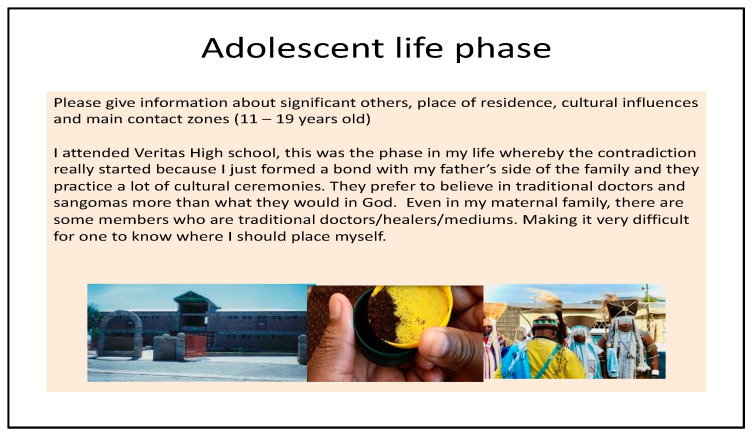
Lihile’s adolescent life phase.

**Figure 4:**
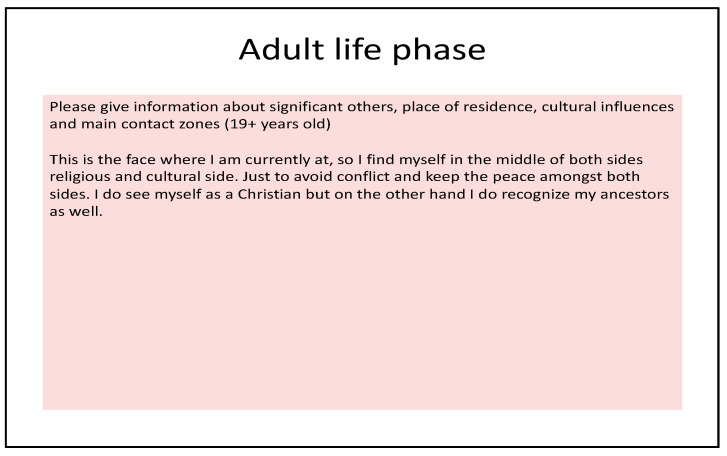
Lihile’s adult life phase.

**Figure 5:**
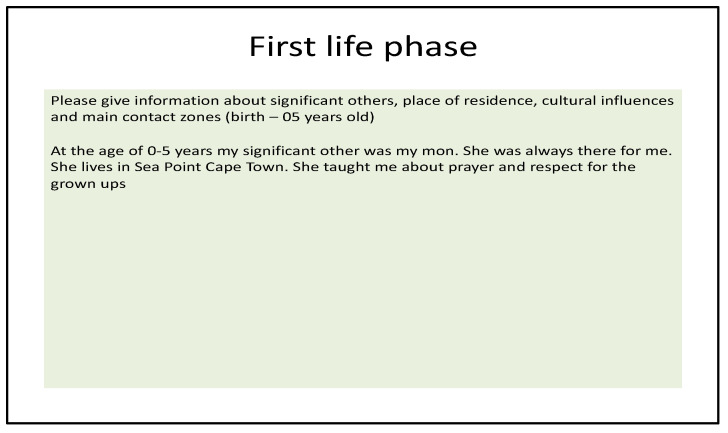
Tanaka’s timeline of her first life phase.

**Figure 6:**
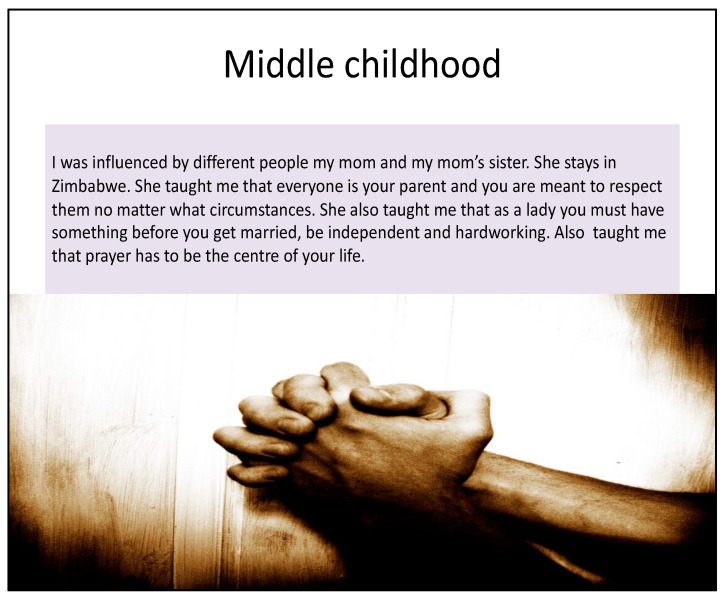
Tanaka’s middle childhood.

**Figure 7:**
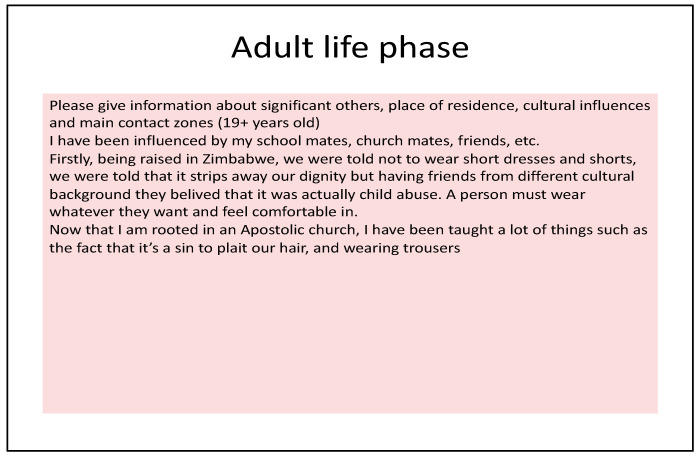
Tanaka’s adult life phase.

**Figure 8:**
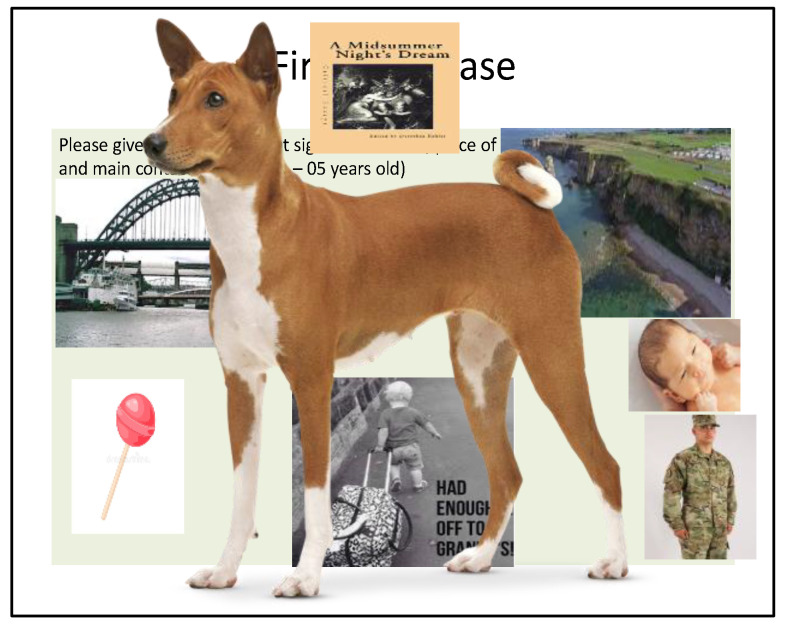
Timeline of Diana’s early childhood.

**Figure 9:**
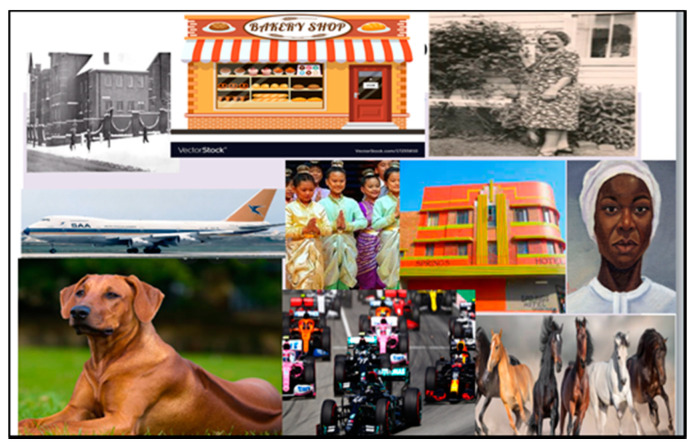
Diana’s timeline for middle childhood.

**Figure 10:**
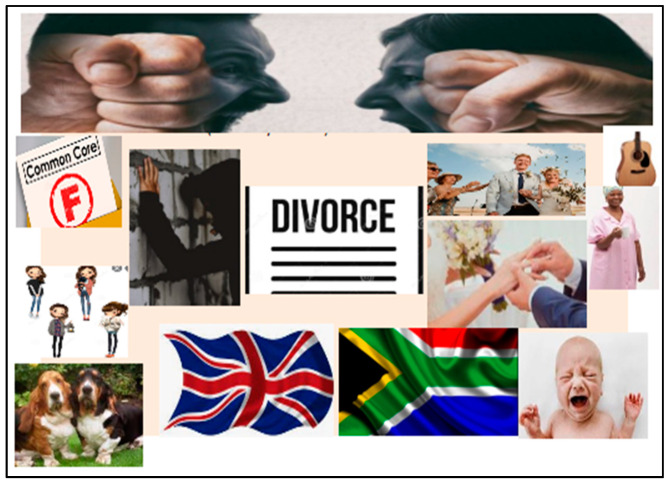
Diana’s adolescent phase.

**Figure 11:**
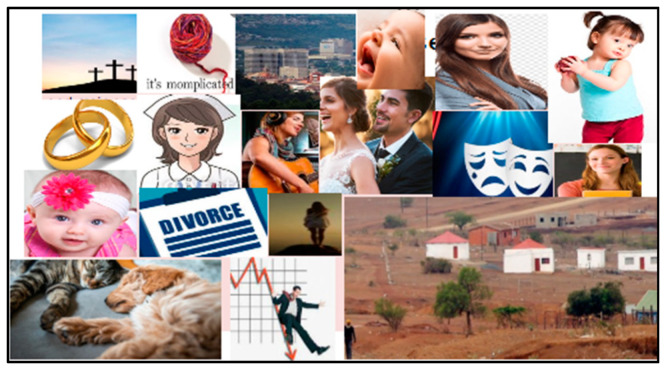
Adulthood in Diana’s timeline.

Photo-elicitation in timeline exercises gave visual and verbal insights into student worldviews, with lived experiences of existential suffering. From an Africentric perspective, Lihile depicted her life in rural SA with colored photographs and text. She described feeling caught in a struggle between Christianity and traditional culture, trying to find peace (Figure 1, Figure 2, Figure 3 and Figure 4). Tanaka also sought a middle path between strong Christian values and traditional and multicultural influences (Figure 5, Figure 6 and Figure 7). Diana’s Eurocentric worldview was challenged in apartheid SA and this made her adaptation to multiple cultures difficult (Figure 8, Figure 9, Figure 10 and Figure 11).

### 3.1. Visual Data

#### 3.1.1. Lihile’s Timeline

Lihile described her difficulty in obtaining photographs of her town as it is so small and remote: “There were a lot of farms, lambs and sheep”. Life revolved mainly around the church, depicted by the Bible, and other cultural influences (Figure 1). However, many of these cultural practices were not allowed to be performed under apartheid. “The apartheid regime didn’t allow a lot of black people to actually perform their cultural ceremonies”. She mentioned that black people were also forced to change their identities to colored to advance in society.

In middle childhood she was conflicted between Christian and traditionalist approaches in her family, describing ceremonies like drinking beer, smoking, “snuif”, and slaughtering sheep as “extreme measures” performed by other black people (Figure 2).

In the adolescent phase of her life, Lihile felt confused by the influence of cultural contradictions when she was exposed to her paternal Sotho family, with “traditional doctors, healers, and mediums”, making it difficult for her to position herself (Figure 3). Finally, in her current adult phase, she viewed herself in the middle of Christianity and traditionalism, trying to avoid conflict and find peace (Figure 4).

#### 3.1.2. Tanaka’s Timeline

Tanaka’s timelines were mostly written texts, revealing a similar focus on the church and her struggle between traditional practices and Christianity (Figure 5). Like Lihile, she received care from her maternal family and learnt strict values and beliefs from the church. Tanaka valued education in her adolescence and was greatly influenced by multicultural school peers and friends. Her only visual image was of hands joined in prayer (Figure 6), with the idea that “prayer has to be the centre of your life”.

#### 3.1.3. Diana’s Timeline

Although Diana’s timelines were filled with bright and graphic imagery and photography depicting a varied life, she revealed dark shadows of neglect and the trauma of living in apartheid SA. She felt that something was wrong: “But as a child you don’t have the maturity or vocabulary to actually express what it is you are sensing”. Her mother, who was creative and artistic, won an art scholarship to Oxford University, but fell pregnant at 16, which prevented her from studying further, and Diana did not feel wanted: “I felt like an imposition, an inconvenience”.

She spent her early childhood in South Shields, NE England, with long walks along Time Bridge with her aunt who bought her lollipops, and she loved the docks and the river. She had a very close relationship to Gaga, her maternal grandmother, whom she felt loved her unconditionally. She sought solace and comfort from Jock, the family dog (Figure 7), with whom she would lie and sob under the stairs when her mother forbade her to visit Gaga.

Middle childhood photographs range from her “Dickensian” school to Gaga’s bakery shop, which was filled with warmth and delicious treats. At nine years her family moved to SA to start a new life, with the Government placing immigrants in the Springs Hotel. Her father pursued Formula 1 racing, while her mother opened a horse-riding school. Diana enjoyed the performing arts and acted in a production of The King and I.

In adolescence she suffered from her father being “unpleasable” and continually asking her: “You don’t think you are a little shit?”, which made her feel not good enough. She was also beaten for her younger sister’s misdemeanors and would be placated by Lettie, her black caregiver:

“They make the most amazing mothers, these black women—they have raised our white children in this country. They have this built-in instinctive maternal acceptance…Lettie laid down her life for us and was even locked up because my mother did not do what was right for her (in renewing her dompas, a travel pass). But her commitment to us children was that she came back”.

This period of her life was marked by her parents partying, drinking excessively, and fighting. Her parents got divorced and her mother moved back to England, where Diana enjoyed reconnecting with her wider family. However, her mother hated it, and they came back to SA. At 16 Diana had a child, who was given up for adoption. Her adult timeline shows a mixed period of rebellion, “dabbling in drugs and alcohol”, a Christian conversion, first marriage, having a daughter, divorce, remarriage, financial struggles, and a move to teaching at a school in a rural area.

### 3.2. Themes

An account of word frequencies was made across all interviews with the three students, covering timeline semi-structured interviews, showed the following codes (Table 2):

Students’ perspectives on well-being in postcolonial SA showed the key developmental role of primary caregivers in the shaping of worldviews and cultural practices in the complex multicultural African context. This conception of the world was further influenced mainly by educational as well as religious CZs. Their struggles to survive the traumatic legacy of social injustices acted as a life force towards well-being and self-transcendence. The main findings—presented as four identified themes—revealed the social embeddedness of functioning well, despite difficulties.

#### 3.2.1. Theme 1: The Key Developmental Role of Primary Caregivers

Primary caregivers and their cultural practices greatly shaped students’ worldviews in a complex multicultural African context. All participants came from female-headed households, with a consistent absence of father/husband support. Kinship care was the term used for caregiving in traditional communities, with the concept of the village raising the child. Support, however, was sometimes punitive and did not extend to emotional healing and educational assistance. Caregiving experiences of injustices were inextricably linked to social atrocities like apartheid and colonialism.

Apartheid affected Lihile’s caregivers gravely as she spoke of absent males working on the farms for long periods and people changing identity to be more socially acceptable:

“We are actually, skin-tone-wise, all mixed. Some are lighter, some are darker—we’re not all the same shade, even our hair is not the same texture {laughs}… So, some members in my family actually transitioned from black to colored—they completely changed the pronunciation of their surnames, their names, everything. I don’t know how the others managed to stay Xhosa over the whole entire time of apartheid”.

Colonialism affected Tanaka, whose early experiences in Zimbabwe resulted in a nomadic life, following her mother from job to job, attending many schools and eventually being taken care of by her aunt, who did not treat her kindly: “My aunt used to provide shelter for me and food as the key supports, but I could not actually talk about … emotional things or other problems. No”. The insecurity of her early childhood left its mark, with issues of lack of self-esteem: “I feel like she (the aunt) actually messed up my self-esteem. Like, wherever you go, whatever your title, whenever you associate with people, you just feel like, ‘I’m not good enough’, because it’s something that has been already instilled in your mind”.

For Diana, moving between England and Africa in childhood and adolescence posed challenges in cultural adaptation. Alienation from her father reflected childhood neglect, with her African caregiver, Lettie, giving her unconditional love after her mother’s beatings:

“Lettie was my first encounter with a person from a different culture and who then became my primary caregiver … [She] basically raised my sister and I. She was always there—she lived in and she’d make sure we were bathed and fed and put to bed, and I was introduced to the Tokoloshe and … and Lettie used to keep us entertained for hours with stories about her culture, her life and her family … She was a Ndebele woman”.

In summary, primary caregiving experiences shaped values and beliefs for all three students. They all suffered neglect and alienation resulting from social atrocities: the colonial and apartheid systems of oppression (Lihile), the colonial system of oppression (Tanaka), and unjust acts of the oppressor (Diana).

#### 3.2.2. Theme 2: The Influence of Contact Zones

Apart from their families, the learning environment was one of the primary CZs for students at school and university. For Lihile, it was not a value instilled by family members, who were mainly farm laborers. Tanaka found schooling difficult due to economic pressures, with little consistency. However, she persisted in her studies and when she attended SA high school, she mixed with many cultures, eventually becoming head girl: “At school, I was made to feel like I’m a good leader with positive influence on people … I became the head girl the following year”. She learnt to abide by her principles and respected the decisions of others, believing that culture sticks with you:

“They used to say we are like ‘salad’… [an acronym for] stupid Africans, following [loving] American [dress] ways, or styles or whatever … So, I feel like we were raised in a Western way. Even though we knew our culture. It was up to us. It … was not forced on us. It was something that we had to decide on …”

She believed that migrating Zimbabweans are being exposed to global influences and that Shona traditions are under threat because people are trying to fit in.

For Diana the transition to SA from England at the age of 8 and then back to England, returning to SA at 16, meant she struggled to fit in academically and emotionally. Despite these different exposures, she excelled in Afrikaans with strict Mr. P., a teacher who terrified her in the days of corporal punishment in SA schools: “He made me stand in front of the class and he held up my book … and I can remember wanting to die a million deaths and he turned around and said, ‘Diana, you’ve got a gold star’ and I burst into tears”.

Diana fell pregnant in SA as an unmarried teenager and was sent away to have the baby and gave it up for adoption. She was not allowed to return to school because of the Calvinistic shame attached: “When I had the baby, I wanted to come back and finish my matric but they wouldn’t let me. They said, half the school would count me a heroine and the other half would hate me. {He was a} very Calvinistic headmaster”.

Sibani [35] explains that the integration of culture in the African context functions as an interrelated whole—although it is useful to structurally break it down into parts. This holistic functioning was clearly expressed by the participants as they spoke about good human relationships operating in harmony with sacred religion. This CZ came in the form of identification with institutional church attendance.

Indeed, religion, as a particular system of faith and worship, played a large part in the lives of all three students. Lihile identified as a primarily Xhosa Christian, with a great grandmother founding the Apostolic Faith Ministries Church active in many parts of Africa. Growing up in a rural Xhosa family implied being tolerant of many Xhosa and Sotho traditions from her mother’s and (later) father’s families respectively. However, she was baptized in a Christian church without the traditional goat-slaughtering ceremony to introduce her to the ancestors, which led to confusion:

“The confusion comes in, especially since I am in the middle of being a Christian and a traditionalist, because now I can’t merge the two beliefs together that I’ve been taught. So now I am in the middle of both, you know”.

Tanaka’s maternal grandparents were Mozambican, but she had no exposure to their heritage. She learnt Shona traditional practices as guided by her Beth Zaida Apostolic Church and followed strict Christian rules as practiced in her faith community into early adulthood (Figure 3). Some values, such as no sex before marriage, were viewed as both traditional Shona and spiritual Christian beliefs. In SA high school she was exposed to multicultural practices and became more tolerant of differences:

Long-term exposure to the church in Africa, representing the CZ for active participation at religious practices, had an immense impact on the worldview of the students, with clear cultural differences. This breaking down of the ‘different’ in regular meetings with fellow-Christians, e.g., Bible study and/or prayer meetings, was articulated by Diana who identified with isiZulu traditions in the following way: “It means like serious regular get-togethers. It means eating sheep’s brains. It means eating chicken feet”.

The struggles and conflict between the cultural influences as experienced by being exposed to various CZs harboring Western/European and African worldviews (Xhosa, Shona, Zulu, Sotho; and Afrikaans, English) also included participants’ experiences of past pain and hindrances. These were related to historical traumas.

#### 3.2.3. Theme 3: Surviving the Legacy of Social Injustices

All students referred to difficulties and hardships that took place during the apartheid regime, entrenching racial inequalities and economic hardship for the majority of South Africans. Living in a rural farming town, Lihile’s family had little social support or opportunities for advancement. The negative influence of postcolonialism was described by Tanaka as difficulties associated with homelessness and poverty.

Diana experienced neglect as a child and sought attachment from her black caregivers, feeling their suffering under oppression, including inhumane practices such as the movement-restricting dompas (travel permit), when caregivers were jailed for non-compliance by neglectful employers. She struggled to accept history lessons, depicting blacks as aliens: “When I was at high school, I just clearly remember being taught that blacks are dirty, blacks are disgusting, blacks are evil, and don’t touch blacks”.

Nevertheless, the narratives of all participants showed many stories/examples about their resilient overcoming and self-transcendence towards well-being and positive functioning, despite exposure to many traumas, including intergenerational and historic. Insights into struggles meant that they sought meaning and acceptance of conflicting worldviews.

#### 3.2.4. Theme 4: Well-Being and Self-Transcendence

Constructs of respect, peace, tolerance, and acceptance came up for all three students. Despite difficulties, they gained self-insight into what it means to live peacefully with different cultures and influences. Positive experiences for Lihile, for example, were mostly articulated in terms of spiritual experiences, church, and religious practices: “So when I walk through that (church) door, I forget about every cultural, every traditionalist influence … I’m pretty committed in that moment, or in those three hours, to whatever is taking place there at that time, or during the week”.

For Tanaka, well-being meant having hope, meeting positive people at school who made her believe in herself. Diana found that Christian values gave her the strength to overcome multicultural challenges in the rural community where she worked: “It means being exposed to different worldviews that are alien to my own. It means becoming more tolerant and accepting, not necessarily agreeing, but agreeing to disagree and find common ground as human beings”.

Their experiences of subjective well-being acted as enablers for participants in their interaction with others within their particular CZs and the richness of integration when accommodating various cultures. Well-being for all three students meant giving back to others in the field of mental health. Lihile wanted to work in the community and focus on the health of others in a non-judgmental way, with total acceptance of both self and others:

“It’s not just about me at the end of the day. It’s about assisting another person as well and not coming across as being prejudiced or judgmental. So, I would put my differences aside and focus on a particular client and try and assist without adding what I believe is right, without implying [imposing] my beliefs on them and making them choose or decide that what I believe is right and what they believe is wrong”.

Tanaka valued respecting others and tolerating differences for positive connection:

“I think, first of all, a person doesn’t have to feel like their culture or their religion is the best and is the one and only that is out there. Because it’s more like churches. We all worship God, but we have different ways of doing it. Some worship under a tree, some in buildings … So, you have to … understand and respect them, respect the differences that you have. We are different, but we are the same”.

Diana wanted SA to hold more Truth and Reconciliation Commissions to improve communication and understanding. She believed in the essence of humanity to go beyond differences to connect with others:

“We are all human; we all coexist on this planet. The fact that you eat different food, get married in a particular way, or give cows to pay labola (bride wealth) has absolutely no bearing on my reaching out to you as a fellow human being”.

## 4. Discussion

This paper aimed to bridge the Afri–Eurocentric worldview divide in postcolonial SA. Because engaging with various contextual cultural practices in Africa is central to an Africentric worldview, students’ exposure to Xhosa, Sotho, Shona, Zulu, as well as English and Afrikaans traditions, was important. It is noteworthy that Atta-Asiedu [6] states that efforts to westernize the African represent the problematical trends of modern assault on African epistemology rather than the de-Africanizing of the African.

Such statements support the findings of this explorative study that the multilayered perspectives of well-being within a multicultural African context embrace both Africentric and Eurocentric worldviews, with enriching experience from the multiple influences.

Firstly, the major effect of students’ primary caregivers’—especially in their developmental years—clearly illustrated the complexity of phenomena related to person-context interaction [19,20]. The social embeddedness of students’ worldviews was fortified with their exposure to highly diverse cultural backgrounds—particularly educational and religious CZs.

The retrospective information about the development of students’ personhood in the African context proved to be valuable to expose similarities. For example, Lihile’s family living in a rural farming town had little social support or opportunities for advancement; Tanaka dealt with difficulties associated with poverty, leading a nomadic life in Zimbabwe as a young child. Diana experienced neglect as a child and sought attachment from her black caregivers, feeling their suffering under oppression.

Despite much intergenerational struggles and historic strain, the narratives of all participants showed many examples of their resilient overcoming and transcending these struggles. According to Wong [1], human beings are hardwired as spiritual beings to survive in a world of dangers and disasters. Insights into struggles meant that participants sought meaning and acceptance of conflicting worldviews as they were confronted with existential issues [1,12]. These insights and resolving of crises to pursue positive goals did not reveal an Africentric worldview free from Eurocentric influences for these students.

Then again, navigating life in multicultural African contexts is an integral aspect of Africentricism. Nyamweru [5] states that being African in the African context entails acknowledging the basic postulates of an African worldview such as influential agents, moral visions, and social processes in the formation of an African personhood [36]. Here the African worldview was different for both black students.

Firstly, for Lihile, an early recollection was confusion with exposure to traditional funeral rites, such as chalk-smeared bodies, burning of herbs, and wearing beads. She had been baptized in the Christian church and these traditional rituals felt alien to her. Still, in her early 20s, she spoke about the deep divide between traditional and Christian practices. When in church, she gave herself fully to the process of prayer, but when at traditional ceremonies with family members, she followed them out of respect but was not wholly committed to the process.

Chawane [18] opines that African perspectives must be the framework for this continent’s knowledge systems; and that we misunderstand Africa when viewpoints and terms other than that of the African to study Africa are used. Then again, African philosophers like Attoe [37] state that thinkers in the African context are aware “… of the existential quagmire” (p. 127) that exists in trying to understand the significance of existence.

Beyers and Mphahlele [38] emphasize that the belief in ancestors is central to African religious practices. They state that earthly descendants (e.g., children, parents, and grandparents) stand in a personal relationship with their ancestors, who have supernatural or sacred status. From a hierarchical superior position to humans, however, ancestors are inferior to God and they are not of divine nature. Overall, ancestors are believed to occupy a higher level of existence than living human beings, bestowing either blessings or illness on their descendants. Seemingly, partaking in these traditional practices to acknowledge and respect ancestors is not shared by all persons living in Africa.

Secondly, for Tanaka, her early childhood in Zimbabwe was seeped in Shona Christian traditions, with her paternal grandmother founding the Apostolic Church to which she belonged. While the importance of culture was acknowledged and accepted, she followed strict Christian rules, such as no sex before marriage and opposition to the ‘evil’ of witchcraft practices. She became more tolerant of cultural differences at SA high school, describing the modernists calling her abused for the strict dress code she followed, such as wearing long skirts, not pants. The traditionalists called the modernists ‘Salads’ (stupid Africans loving American dress): “Yeah, it was everything it’s not just the dressing, but even the culture, you know … like being cheesy”.

This perspective can be aligned with Rabaka [2005, in 6] stating that the African worldview theory is “… essentially a combination of the classical and contemporary, continental and diasporic African overarching outlook on human experience” (p. 1). Atta-Asiedu [6] clarifies that the African worldview is unique, specifically grounded and growing out of local history and culture. Its essence is undeniable and not inferior to, for example, Eurocentric perspectives.

Thabede refers to Reve who stated in 1995 that “… the word African has been debatable… [as] many people also claim to be Africans. This has created real confusion as to what an African is” [39] (p. 233). African in this context refers to African (black) people who are residents of SA and fall within the Zulu, Xhosa, Sotho, Tsonga and Venda ethnic groups. This means that African excludes, for example, the white, colored, and Indian populations residing in SA [39].

Thirdly, Diana, born in England and growing up in Africa, represented the Eurocentric worldview. While typically ‘colonial’, she adapted to the Afrikaner culture. She had an estranged relationship with her parents but occasionally discussed injustices of the apartheid system with her father. She reflected on the complexity of working in rural schools in KZN by stating that “it means being exposed to different worldviews that are alien to my own”.

She also observed that English was sometimes the preferred choice of communication amongst the isiZulu population where she lived. This reality of black children speaking English versus solely their mother tongue could be seen as the dropping of African values and African people being westernized [6]. This is not uncommon in postapartheid SA “… where experiences are influenced by highly diverse and hybrid cultural and language backgrounds” [31] (p. 130). So, since socialization is of key importance for the transmission of social norms and traditional practices, it seems that various adaptations take place regarding the language of cultural practices and even family rituals [40].

The dealing with intercultural conflict in SA CZs was expressed by all participants. These conflicts were an integral part of their narrations, especially spiritual practices related to religious ceremonies of these church-going students and rituals characteristic to traditional African practices performed by, for example, sangomas. All students had to cope with differences and sensitive issues associated with traditional and non-traditional upbringing. These conflicts occur due to multiple intersectionalities, such as language and value sets and experiences of inequality, power struggles and disadvantages related to poverty [31].

Being exposed to various CZs is closely associated with the key role of influencers in the shaping of individuals’ worldviews. Mayer et al. [31] describe that these are created “in the context of colonial encounters” (p. 130) where humans interact. In our global village, these CZs are spaces where individuals and groups form “intercultural relations” (p. 130) by means of cultural and transcultural exchange. In the current study these CZs were expressed as mostly constructive intercultural spaces of families, faith communities/church and the learning environment.

Although the integration of these CZs is not foreign to the African context, Mayer et al. [31] clarify that they exist due to global and local migration flows and, as transcultural spaces that “…create habitats of shared social knowledge and practice, power and identity—all concepts which are renegotiated around the dominant culture” (p. 131).

Sibani [23] refers to aspects as language, attitudes, morals, values, habits, philosophy, knowledge, principles, and conventions as the non-material manifestations of culture. These, with material tangible objects such as visible products like cooking utensils, form the totality of the way of life of a people [23].

Surviving the legacy of injustices of colonial and racist legislation revealed past practices of gross human violation. All participants expressed ‘old pain’, but the quality of these experiences differed.

For Lihile, intergenerational trauma was transmitted at family gatherings when unrecognized family members who were so-called colored, arrived as strangers, but little was spoken about this unjust history. She overcame apartheid-inflicted poverty and social injustices in her Xhosa family and despite confusion and mixed exposure to different traditional and spiritual practices, she navigated a space of tolerance and acceptance.

Tanaka suffered insecurities and lack of self-esteem during her neglectful Shona childhood, struggling with no social support in postcolonial Zimbabwe. She was able to find tolerance of others in her strong spiritual values, finding hope and belief in herself to pursue her academic and personal goals of fulfilment.

Diana gave insights into the cultural complexity of the impact of Eurocentricity in Africa, finding wellness through deep spirituality and respect for others, despite differences. While cultural diversity is commonplace to the African context, embracing the difficulties associated with different cultures entails bravery to overcome multiple issues. These include family fragmentation, the conflict of highly diverse and hybrid cultural and language backgrounds [40], as well as the serious limitations of past and present social injustices.

The final theme revealed the psychofortology of attaining well-being and transcending past and present challenges amidst the complexity of multicultural influences. Insights guided by the eudaimonic approach typical to PP look beyond demographical challenges and set apart the diverse social mechanisms [40] to embrace feeling and functioning well. While the grave difficulties associated with lack and the negative spirals of inherited poverty and injustices should not be ignored, this paper showed the positive power and significance of interconnectedness, which can vary based on cultural context and life situatedness [41].

Well-being is intricately interwoven with spirituality [1]. Knowledge of the Supreme Being is central to the worldview of the traditional African, which does not require a deep and formal theology for people to flourish [37]. Jacob Olupona’s (African professor of indigenous African religions at Harvard Divinity School) showed that, for many Africans, religion is a way of life that informs everything in traditional society. This perspective echoes Attoe’s [37] understanding that indigenous African spirituality does not represent a form of theocracy or religious totalitarianism, but it simply acknowledges that beliefs and practices inform every facet of human life.

In summary, each student suffered from different experiences of injustices. Through the dual lens of existential suffering and indigenous understanding, each found the healing gold in their life but it was also a struggle, with unspoken pain and confusion. All participants referred to values of respect, tolerance, and acceptance to live peacefully amidst different cultures and influences. The culmination of personal, relational, and collective strengths [42] offered the overcoming of risks of multicultural encounters to add value to Africa and its people [43]. In this sense, human beings living under the African sun are indeed made in Africa [6].

Finally, while rich information was gathered, the small number of participants and the lack of gender diversity must be mentioned as limitations of the current study. Also, since the collection of visual data (photo-elicitation in retrospective timelines) contributed greatly to a better understanding of worldviews, the lack of pictures provided by Tanaka is acknowledged as a limiting factor. The use of art/visual material and narratives is recommended in the exploration of worldviews in future research.

## 5. Conclusions

This small-scale study showed that despite widely differing perspectives of Africentric and Eurocentric worldviews, the lived experiences of students overcoming suffering in SA have many commonalities in pathways to well-being. Evidently, both suffering and flourishing are integral to living well.

It became clear that the transcendence of existential pain through caregiving, learning, interconnectedness, and spirituality were key components in their well-being. Students’ positive goals included the reaching out across racial and economic divides to work in the healing and teaching professions. They were willing to take on the multiple challenges of Africa, attempting to overcome past injustices and traumas experienced in this troubled continent. Belonging to a global generation, they expressed hope for the future, deeply affected by traditions, but also benefitting from Eurocentric influences to deal appropriately with healing processes. While past atrocities needed to be integrated, they sought reparation in future healing work.

Although students stood on different sides of the worldview divide with varying experiences of pain and suffering, they shared commonalities of being human and were able to find bridging steps to well-being. The focus on individual and collective wellness is, indeed, part of the healing process not only for the oppressed but also for the oppressors of past social injustices.

This research was an attempt to transcend the divide of Afri–Eurocentric worldviews towards a shared responsibility to develop an improved social science in Africa. The African colonial caged mind can, indeed, be set free to soar, benefitting from global influences to reach new heights of knowledge and insights.

## Figures and Tables

**Table 1:** Demographics of student participants.

Participants	P1	P2	P3
Name	Lihile	Tanaka	Diana
Gender	Female	Female	Female
Age	23	24	62
Marital status	Single	Single	Married
Children	None	None	Three
Religion	Christian	Christian	Christian
Name of church/ faith community	Apostolic Faith Ministries	Apostolic Faith and Mission Church	Newborn Revival Church
1st language	Xhosa	Shona	English
2nd language	English	Xhosa	IsiZulu (101)
3rd language	Afrikaans	English	Afrikaans
Country of birth	South Africa	Zimbabwe	England
Place of birth	De Aar	Murehwa	South Shields County, Durham
Schools attended	One primary school; One secondary school	Five primary schools; Three high schools	One junior school in England; a primary school and two high schools in Gauteng
Current studies (2021)	Third year BA	Third year BA	BA Honors
Major/s	Psychology	Psychology	Counseling communication
Plans for future	Study postgrad; work in a mental health field	Study Honors; teach English	Trauma counseling by means of dramatic arts

**Table 2:** Examples of word count with references (NVivo 12).

Word	Count	Example
Family	131	“I was actually raised by my grandparents on my mother’s side of the family”. (Lihile)
Mother/ mum/mom/ sister/grandmother and maternal	324	“Up to the age of five, I was living with my mom … She was the one person who was mostly influential to me because I was always with her”. (Tanaka) “Growing up, my neighbor would actually discipline me—like beat me—if she saw me doing something that was wrong … And my mom was actually okay with that … So it’s more like, it takes a village to raise a child. (Tanaka)
Dad/paternal/ husband	82	“From my father’s side of the family, I never really had a bond with him—when I was born until I was a teenager. (Lihile)
Culture/ cultures/cultural	150	“The culture I was born into was bohemian, eclectic, and arty. My grandparents were cosmopolitan and liberal”. (Diana)
West/ern/ westernized	66	“I struggle with it, because being a black person, you are actually told that everything, … who you are, comes actually from your ancestors, your roots. And now with the Western side, it’s almost as if: ‘Who do I believe now?’ because this is actually theory, and the other one is your history”.(Lihile)
Africa/South African (SA)	114	“If I have a child who goes to school in SA, he will be exposed to many cultures. Because of these experiences in this country he’s more likely to drift from the Shona culture to becoming whatever culture he is exposed to”. (Tanaka)
Shona	52	“Firstly, I think it’s important to teach your child about their roots, where they come from. I’ll probably teach my child about the rules, the Shona culture, everything, everything about Shona, including the language”. (Tanaka)

## References

[1] Wong P.T.P. (2020). Second wave positive psychology’s (PP 2.0) contribution to counselling psychology. Couns. Psychol. Q..

[2] Nwoye A. (2015). What is African psychology the psychology of?. Theory Psychol..

[3] Viljoen H., Painter D., Moore C., Viljoen H.G., Meyer W.F. (2017). African Perspectives. Personology—From Individual to Ecosystem.

[4] Naidoo A.V. (1996). Challenging the hegemony of Eurocentric psychology. J. Community Health Sci..

[5] Nyamweru C. (2016). How to Put the ‘African’ Back into African Studies. African Arguments. https://www.africanarguments.org.

[6] Atta-Asiedu K.A. (2020). African Worldviews and Research in African Studies: The Missing Ingredient. SSRN Electron. J..

[7] Kelland M.D. (2020). The African Worldview and Spirituality. Lansing Community College. https://socialsci.libretexts.org/@go/page/12279.

[8] Ebersöhn L., Loots T., Mampane R., Omidire F., Malan-Van Rooyen M., Sefotho M., Nthontho M. (2018). An indigenous psychology perspective on psychosocial support in Southern Africa as collective, networking, and pragmatic support. J. Community Appl. Soc. Psychol..

[9] Nyoni J. (2019). Decolonising the higher education curriculum: An analysis of African intellectual readiness to break the chains of a colonial caged mentality. Transform. High. Educ..

[10] Hübl T., Avritt J.J. (2021). Healing Collective Trauma.

[11] Statistics South Africa [Stats SA] Yearly Archives: 2021. https://www.statssa.gov.za/?m=2021.

[12] Wong P.T.P. (2013). Toward a dual-systems model of what makes life worth living. The Human Quest for Meaning: Theories, Research, and Applications.

[13] Chang E.C., Downey C.A., Hirsch J.K., Lin N.J. (2016). Positive Psychology in Racial and Ethnic Groups: Theory, Research, and Practice.

[14] Jans-Beken L., Wong P.T.P. (2021). Development and preliminary validation of the Existential Gratitude Scale (EGS). Couns. Psychol. Q..

[15] Naidoo L., Van Schalkwyk I. (2021). Pathways to academic success of disadvantaged undergraduate university students from a high-risk community in the Western Cape. S. Afr. J. High. Educ..

[16] Abdulla M.R. (2018). Culture, religion and freedom of religion or belief. Rev. Faith Int. Aff..

[17] Berkessel J.B., Gebauer J.E., Joshanloo M., Bleidorn W., Rentfrow P.J., Potter J., Gosling S.D. (2021). National religiosity eases the psychological burden of poverty. Proc. Natl. Acad. Sci. USA.

[18] Chawane M. (2016). The development of Afrocentricity: A historical survey. Yesterday Today.

[19] Bronfenbrenner U. (1979). The Ecology of Human Development: Experiments by Nature and Design.

[20] Rosa E.M., Tudge J. (2013). Urie Bronfenbrenner’s Theory of Human Development: Its Evolution from Ecology to Bioecology. J. Fam. Theory Rev..

[21] Mahali A., Lynch I., Fadiji A.W., Tolla T., Khumalo S., Naicker S. (2018). Networks of Well-being in the Global South: A Critical Review of Current Scholarship. J. Dev. Soc..

[22] Philipps J. (2018). A Global Generation? Youth Studies in a Postcolonial World. Societies.

[23] Sibani C.M. (2018). Impact of Western culture on traditional African society: Problems and prospects. J. Relig. Hum. Relat..

[24] Yin R.K. (2014). Case Study Research Design and Methods.

[25] Glaw X., Inder K., Kable A., Hazelton M. (2017). Visual Methodologies in Qualitative Research: Autophotography and Photo Elicitation Applied to Mental Health Research. Int. J. Qual. Methods.

[26] Kara H. (2017). Creative Research Methods—Arts-Based Methods (Part 1 of 3). National Centre for Research Methods. https://www.youtube.com/watch?v=7PgWTVL92RM.

[27] Harper D. (2002). Talking about pictures: A case for photo-elicitation. Vis. Stud..

[28] Adriansen H.K. (2012). Timeline interviews: A tool for conducting life history research. Qual. Stud..

[29] Cleland J., MacLeod A. (2021). The visual vernacular: Embracing photographs in research. Perspect. Med. Educ..

[30] Maree J.G., McMahon M., Watson M. (2015). The Early Recollections Technique. Career Assessment.

[31] Mayer C.-H., Makhura R., Akii A., Dateling T., Dineo P., Ebrahim T., Jordaan E., Khoza K., Mabanya C., Mpatane A. (2021). Narrations on intercultural experiences in South African contact zones. Int. J. Intercult. Relat..

[32] Braun V., Clarke V. (2013). Successful Qualitative Research—A Practical Guide for Beginners.

[33] Creswell J.W. (2014). Research Design: Qualitative, Quantitative and Mixed Method Approaches.

[34] Lincoln Y.S., Guba E.G. (1985). Naturalistic Inquiry.

[35] Berger R. (2015). Now I see it, Now I don’t: Researcher’s Position and Reflexivity in Qualitative Research. J. Qual. Res..

[36] Appiah R., Wilson-Fadiji A., Schutte L., Wissing M.P. (2020). Effects of a Community-Based Multi-component Positive Psychology Intervention on Mental Health of Rural Adults in Ghana. Appl. Psychol. Health Well-Being.

[37] Attoe A.D. (2020). A systematic account of African conceptions of the meaning of/in life. S. Afr. J. Philos..

[38] Beyers J., Mphahlele D.N. (2009). Jesus Christ as ancestor: An African Christian understanding. HTS Teol. Stud./Theol. Stud..

[39] Thabede D. (2008). The African worldview as the basis of practice in the helping professions. Soc. Work/Maatskaplike Werk.

[40] Wissing M.P., Wilson Fadiji A., Schutte L., Chigeza S., Schutte W.D., Temane Q.M. (2020). Motivations for Relationships as Sources of Meaning: Ghanaian and South African Experiences. Front. Psychol..

[41] Wissing M.P. (2014). Meaning and relational well-being in cross-cultural perspectives. J. Psychol. Afr..

[42] Prilleltensky I. (2019). Mattering at the Intersection of Psychology, Philosophy, and Politics. Am. J. Community Psychol..

[43] Chigudu D. (2018). Strength in diversity: An opportunity for Africa’s development. Cogent Soc. Sci..

